# Preoperative intestinal stent decompression with primary laparoscopic surgery to treat left-sided colorectal cancer with obstruction: a report of 21 cases

**DOI:** 10.7497/j.issn.2095-3941.2013.02.006

**Published:** 2013-06

**Authors:** Chao Zheng, Yu-Lian Wu, Qing Li

**Affiliations:** 1 Department of Gastrointestinal Surgery, The First Affiliated Hospital of Zhejiang Chinese Medicine University, Hangzhou 310006, China;; 2 Department of General Surgery, The Second Affiliated Hospital of Zhejiang University Medical School, Hangzhou 310009, China

**Keywords:** Colorectal cancer, intestinal obstruction, intestinal stent, laparoscope

## Abstract

**Objective:**

This work aimed to study the safety and efficacy of preoperative intestinal stent decompression combined with laparoscopic surgery to treat left-sided colorectal cancer with obstruction (LCCO).

**Methods:**

Retrospective analysis was conducted on data obtained from 21 LCCO patients admitted to The First Affiliated Hospital of Zhejiang Chinese Medicine University during March 2008 and December 2011. To remove the intestinal obstruction, preoperative intestinal stent placement under colonoscopic guidance was performed. Approximately 7 to 10 days after the operation, laparoscopic radical surgery of colorectal cancer was conducted.

**Results:**

Among the 21 cases studied, laparoscopic surgery was successful in 20 patients. Emergent laparotomy was conducted in one patient because of tumor invasion in the ureter. The duration of the operation ranged from 180 to 320 min, and the average time was 220 min. The recovery time for bowel function ranged from 2 to 5 days with an average time of 3 days. Postoperative infection of the incision occurred in one case. No anastomotic leakage was observed in any of the cases.

**Conclusion:**

Preoperative intestinal stent decompression, combined with primary stage laparoscopic surgery, is a safe and effective method for the treatment of LCCO.

## Introduction

Left-sided colorectal cancer develops slowly and unnoticeably because of its anatomic, psychological, and physiological characteristics. Symptoms of intestinal obstruction are the common complications at the middle and late stages of left-sided colorectal cancer. For patients with such conditions, traditional surgery involves the Hartmann operation and further treatment by stage II operation. After a long treatment cycle with two surgeries, the patient slowly recovers and experiences considerable spiritual and economic pressure^1^. The Gastrointestinal Surgery Department of The First Affiliated Hospital of Zhejiang Chinese Medicine University treated 21 patients suffering from left-sided colorectal cancer with obstruction (LCCO) during March 2008 and December 2011. An enteric stent was placed under the colonoscope to remove the intestinal obstruction, thus avoiding stage I enterostomy. After a comprehensive preoperative examination and bowel preparation, patients received radical resection of the left colorectal cancer under a laparoscope. This procedure is characterized by a small wound, fast recovery, and satisfactory results. The subsequent sections of the paper provide the report.

## Patients and methods

### Clinical data

The patients were composed of 13 males and 8 females within the age bracket of 56 to 81 years with an average age of 67.4 years. The patients had an obstruction period of 12 to 672 h. The patients have been suffering from different degrees of stopping gas passage and defecation, abdominal distention and pain, nausea and vomiting, and other obstructive symptoms during their hospitalization. Different degrees of intestinal dilation and gas-fluid level could be observed by using an abdominal plain radiograph. With abdominal CT, the possibility of left colorectal malignant cancer was considered. Thereafter, an enteric stent was placed under the colonoscope to confirm the pathological diagnosis of colorectal cancer. Six patients with complete obstruction and 15 patients with incomplete obstruction were observed (the diameter at the narrow place of the enteric cavity caused by the tumor was approximately 2 to 10 cm, and the colonoscope could not pass through the narrow cavity). A total of 6 patients were diagnosed with upper rectal carcinoma, 9 patients with sigmoid colon carcinoma, 4 patients with carcinoma of the descending colon, and 2 patients with transverse splenic flexture cancer. Seventeen patients also had chronic internal diseases, such as high blood pressure, diabetes, and cardiopulmonary diseases. This study was approved by the Ethics Committee of The First Affiliated Hospital of Zhejiang Chinese Medicine University. Written informed consent was obtained from all patients.

### Methods

For the preoperative preparation, the patients underwent abrosia to reduce gastrointestinal pressure and correct the water, electrolyte, and acid-base equilibrium. The patients also received nutritional support. The patients underwent abdominal flat radiograph, abdominal CT, and other modes of inspection. The possibility of LCCO was considered.

Preoperative inspection was conducted prior to the placement of the intestinal stent. The intestinal stent was then placed to eliminate intestinal obstruction and obtain the pathological diagnosis. Once the colonoscope reached the narrow section, the angiography catheter was inserted into the narrow section through the endoscopic forceps. The water-soluble contrast agent cardiografin was inserted, and the size, shape, and length of the narrow section were observed by X-ray. The stent transport system was then inserted into the narrow section through the wire, and was observed through X-ray to determine whether the two ends of the stent went beyond the narrow section by 2 cm. Subsequently, the stent was released with its position and opening degree followed closely by colonoscopy and X-ray. The correct stent position under the colonoscope was also observed. All patients had abrosia and stayed in bed on the day of operation.

After the operation, the patients received antibiotic injection to prevent infection, and treatment to suppress acidity and stop the bleeding, and also obtained nutritional support. Patients were closely observed to check whether they had any abdominal pain, hemafecia, defecation, and gas passage. If the patient was still unable to pass gas or defecate within 24 h, the patient could be given intestinal radiography again to follow closely the position and obstruction of the stent and doctors could make necessary adjustments.

### Radical resection under the laparoscope

Intestinal obstruction symptoms disappeared within 7 to 10 d after the intestinal stent was placed. After a complete preoperative inspection and thorough bowel preparation, the patients underwent operation under the laparoscope: (1) radical resection of the rectum and sigmoid carcinoma: the patient received tracheal intubation and general anesthesia at the lithotomy position with the head low and feet raised and inclined to the right. A puncture measuring 1 cm was made along the superior order of the umbilical, and an optical viewer was placed. Thereafter, a 1.2 cm puncture was made at the McBurney point of the lower right quadrant as the main operation hole. A 0.5 cm auxiliary operation hole was made along the navel of the right quadrant and an inverse McBurney point at the lower left quadrant. The pneumoperitoneum pressure was 10 to 12 mmHg. The side peritoneum was then cut open along the left paracolic sulci to expose the ureter and was further disassociated downward along the presacral space. The lymph glands were cleaned at the root of the blood vessels under the mesentery and were severed afterward. Similarly, the intestinal canal was severed 2 cm below the tumor. A small cut was made on the lower left quadrant and inside the abdomen. The near-end intestinal canal of the tumor was pulled out from the abdominal cavity and then severed to remove the tumor. The severed end of the intestinal canal was placed back into the abdominal cavity, and anastomosis was conducted on the far end of the intestinal canal; (2) radical resection of the descending colon and colon spleen carcinoma (left colectomy): the standing position and operation hole of the patient are similar to that of the radical resection of the rectal carcinoma. The side peritoneum was incised along the left paracolic sulci. Subsequently, the descending colon and colon spleen area were disassociated, thus the left colic artery was severed. The regional lymph nodes were then cleaned. A 5 cm opening was made on the left abdomen, the colon was pulled out, and the intestinal canal was severed at 10 cm above and below the tumor. Finally, the biofragmentable anastomosis ring was placed at the end anastomosis of the intestinal canals.

## Results

Obstructive symptoms disappeared within 7 to 10 d after the enteric stents were placed in all 21 patients. Radical resection of the colorectal cancer was conducted under the laparoscope. Under the laparoscope, 20 patients underwent successful operation. One patient underwent laparotomy after the tumor infiltrated the left ureter. No samples of the cut edges have any tumor residue or infiltration. The operation lasted for 180 to 320 min at an average of 220 min. The postoperative bowel function took 2-5 days to recover (3 days on average). One patient had an infection on the cut but was successfully treated through conservative methods and did not produce any stomal leaks. The postoperative hospitalization period ranged from 7 to 14 days (10.5 days on average). In addition, 18 patients received Folfox (Oxaliplatin + 5-fluorouracil + calcium folinate) chemotherapy. The hospital followed up the 19 patients for 5 to 48 months (24 months on average). However, the hospital was unable to follow up two patients: one patient was found to have a metastatic lesion in the liver during the one-year postoperative reexamination and received a second operation; the other patient was found to have an extensive metastasis of the cancer in the abdominal cavity during a postoperative reexamination 18 months after the treatment. Thus, the second patient could not receive a resection again. The second patient died 20 months after the operation. The other patients recovered properly without recurrence or metastasis of cancer.

## Discussion

Approximately 850,000 new patients are diagnosed with malignant neoplasm of the rectum across the world each year, and 7% to 29% of these patients demonstrate the initial symptoms of acute complete or incomplete intestinal obstruction^2^. Given that colorectal cancer is accompanied with obstruction, such patients cannot undergo preoperative bowel preparation. Clinical treatment is also difficult because stomal leak, serious infection, and other syndromes can easily occur after the operation. Nevertheless, preoperative bowel preparation is the most difficult task in the treatment of colorectal obstruction. Local and foreign scholars have developed numerous methods to reduce the pressure and lavage of the near-end intestinal canal^3^^-^^5^, including intraoperative lavage of the intestinal canal, temporary near-end colostomy, intraoperative reduction of pressure by anus intubation, and postoperative placement of the anal canal to reduce pressure. The above methods reduce the occurrence of stomal leak after stage I anastomosis for left colorectal obstruction, and are promoted as clinical treatments. However, these methods exhibit disadvantages, long operation time, abdominal cavity contamination, and electrolyte loss in the intestinal canal, which disturbs the internal environment. In recent years, an increasing number of local and foreign studies have reported the use of metal stent as a support inside the intestinal canal to cure colorectal malignant obstruction^6^^,^^7^. Such an approach can cure the malignant obstruction caused by colorectal cancer permanently or temporarily and allow an operation to be performed at a selected date^8^. The growing popularity of minimally invasive surgery and fast rehabilitation has increased the acceptance of the radical resection of colorectal cancer under laparoscope. The hospital uses a preoperative intestinal stent to reduce the pressure and implements the phase I operation under the laparoscope to cure LCCO and alleviate obstruction. After a comprehensive preparation, which includes intestinal canal cleaning, water and electrolyte regulation, acid-base balancing, and nutritional support, the patients underwent radical resection under laparoscope to avoid the traditional operation by stages and implement a minimally invasive surgery and with fast recovery.

The application of enteric stent for the treatment of colorectal obstruction is divided into temporary transitional placement and palliative treatment^9^^,^^10^. Palliative treatment is suitable for removing obstructions in patients with primary occurrence or recurrence of colorectal cancer in a non-removable local legion with an extensive metastasis or inability to receive tolerance surgery. This treatment avoids the need to bear an anal bag for a long period, thus improving the life quality of the patient. For patients in this group, enteric stents are employed as transitional placements to substitute colostomy, fully reduce the intestinal pressure, and alleviate the obstructive symptoms, thus restoring the local and full-body pathological and physiological state of patients with LCCO into a non-obstructive state. Consequently, patients undergo pure stage I operation under laparoscope for colorectal cancer to reduce the operative syndrome and death rate, thus avoiding the wound of the secondary operation, increasing the survival rate, and improving the life quality of patients. Complications of stent placement mainly include intestinal perforation, intestinal bleeding, stent displacement, stent dislocation, and obstruction^11^^,^^12^. Such complications are primarily related to the operating skills, tumor position and shape, operative instrument, and stent type. For this group, 21 patients received successful operations by experienced endoscopy doctors without intestinal perforation, bleeding, or other complications.

A review of existing studies^13^^-^^15^ shows that colorectal resection under laparoscope is technically safe. The radical treatment of the tumor, length of operation, recent and future effect, postoperative local recurrence, far end metastasis, and survival rate are similar to that of the traditional laparoscope. Operation under a laparoscope has evident advantages in terms of the length of cut, dosage of painkillers, postoperative recovery of intestinal functions, and days of hospitalization. In addition to the enhancement of operative skills, strengthening of the no-tumor concept, and application of new apparatus, the radical resection of colorectal cancer is not only achieved in principle but also delivers better results compared with laparotomy.

In summary, employing the preoperative placement of intestinal stent and stage I operation to cure LCCO under a laparoscope is a safe and effective method that has incomparable advantages over traditional surgery. Thus, the preoperative placement of intestinal stent is worthy of extensive clinical application.
